# Maternal laparotomy to fetoscopic myelomeningocele repair in patients with Class 2 obesity is safe and feasible

**DOI:** 10.1002/pmf2.70078

**Published:** 2025-08-14

**Authors:** Braxton Forde, Charles B. Stevenson, Foong‐Yen Lim, Mounira Habli, David Nelson McKinney, Kara Beth Markham, Mallory Hoffman, K. Schuyler Nissen, Erinn Goetz, Karin Bierbrauer, Jose Luis Peiro

**Affiliations:** ^1^ Department of Obstetrics and Gynecology University of Cincinnati College of Medicine Cincinnati Ohio USA; ^2^ Cincinnati Fetal Care Center Cincinnati Ohio USA; ^3^ Department of Neurosurgery Cincinnati Children's Hospital Cincinnati Ohio USA; ^4^ Department of Pediatric Surgery Cincinnati Children's Hospital Cincinnati Ohio USA; ^5^ TriHealth Obstetrics and Gynecology Cincinnati Ohio USA

**Keywords:** fetal surgery, fetal therapy, fetoscopic surgery, myelomeningocele, obesity

## Abstract

**Background:**

There is minimal to no data on fetal surgery outcomes in the setting of body mass index (BMI) ≥ 35. Theoretical concerns exist for increased maternal risks from surgery and increased surgical difficulty due to maternal body habitus.

**Objective:**

To evaluate the pregnancy and operative outcomes with maternal laparotomy to 3‐port fetoscopic myelomeningocele (MMC) repair in the setting of pre‐pregnancy BMI 35–40 compared with pre‐pregnancy BMI < 35.

**Methods:**

This was a cohort study at a single maternal fetal care center in which maternal laparotomy to 3‐port fetoscopic surgery for MMC was performed. Criteria for offering surgery were same as the Management of Myelomeningocele Study (MOMS) Trial with the exception that, starting in 2022, our center's BMI criterion was increased to include pre‐pregnancy BMI ≤ 40. The primary outcome was the gestational age of delivery after surgery. Secondary outcomes planned were operative times, birthweight, and postoperative maternal and fetal complications. Evaluation of non‐inferiority was planned on the primary outcome and operative times, with a < 1 week decrease in gestational age at delivery in the setting of repair with BMI ≥ 35 being considered non‐inferior.

**Results:**

From January 2022 to December 2024, there were a total of 38 pregnancies that both underwent maternal laparotomy to fetoscopic MMC repair and delivered prior to December 2024. A total of 12 patients had a pre‐pregnancy BMI 35–40. Patients were similar regarding pregnancy characteristics. Regarding the primary outcome, there was no difference in gestational age at delivery between groups (36 2/7 weeks, IQR 34 3/7, 37 5/7 in BMI 35–40 vs. 35 2/7 weeks, IQR 31 4/7, 37 4/7 in BMI < 35, *p* = 0.257). The primary outcome was determined to be non‐inferior with a 95% CI (34 2/7 to 37 6/7), being not inclusive of 34 3/7 minus 1 week (pre‐specified acceptable difference). While times for the fetoscopic surgery were similar between groups, there was a significant increase in operative time of the non‐fetoscopic portions of the case with BMI 35–40 (80 min, IQR 63, 91 vs. 59 min, IQR 55, 71, *p* = 0.047) and non‐inferiority could not be established for overall operative times. However, this did not translate to increased postoperative complications, as other postoperative factors were consistent between groups.

**Conclusion:**

Maternal laparotomy to fetoscopic MMC repair patients with BMI 35–40 is non‐inferior to that of patients with BMI < 35. With the exception of a slightly longer non‐fetoscopic surgery time, the characteristics and pregnancy outcomes are similar between groups. BMI 35–40 should not by itself be considered an absolute contraindication to in utero MMC repair.

## 1 INTRODUCTION

Since the publication of the Management of Myelomeningocele Study (MOMS) [1], there has been growing popularity and interest in prenatal surgery for in utero repair of myelomeningocele (MMC). The inclusion and exclusion criteria are listed in Box 1. A maternal body mass index (BMI) ≥ 35 was chosen as a contraindication to surgery due to the perceived increased surgical complexity secondary to maternal body habitus and the potential increased risk of maternal complications secondary to a possibly longer operative time and/or perioperative tocolytic regimens. As the years have passed since MOMS, a few investigations have been undertaken looking at the expansion of open fetal surgery for MMC in the setting of BMI ≥ 35; however, the data remains sparse. A recent study by Bonanni et al. [2, 3, 4] reviewed three studies of maternal laparotomy to open fetal surgery for MMC repair, concluding that, while significant differences haven't been identified, the level of evidence was low. This highlights the significant limitations in our understanding of fetal surgery in patients with Class 2 or even 3 obesity. This is particularly an issue given the longstanding knowledge that obesity is associated with risks for fetal spina bifida and multiple other congenital anomalies [5, 6, 7, 8].

**BOX 1: Management of myelomeningocele inclusion/exclusion criteria and our current inclusion/exclusion criteria**

**Table:** 

MOMS Trial Inclusion/Exclusion	Our fetal center's inclusion/exclusion as of January 2022
Inclusion ‐ Singleton pregnancy ‐ MMC/myelocele with upper boundary between T1‐S1 ‐ Evidence of hindbrain herniation ‐ Gestational age at randomization of 19.0–25.9 weeks ‐ Normal karyotype ‐ US resident ‐ Maternal age of at least 18 ‐ Ability to relocate to the center ‐ Completed psychosocial evaluation	Inclusion ‐ Singleton pregnancy ‐ MMC/myelocele with upper boundary at T1 or lower ‐ Evidence of hindbrain herniation ‐ Gestational age at repair < 26 weeks* ‐ Normal FISH ‐ US resident ‐ Maternal age of at least 18 ‐ Ability to relocate to the center ‐ Completed psychosocial evaluation
Exclusion ‐ Anomaly unrelated to the MMC ‐ Severe kyphosis ‐ “Risk of preterm birth” including short cervix and prior preterm birth ‐ Placental abruption ‐ Prior hysterotomy in active segment ‐ Current HIV ‐ BMI ≥35 ‐ Other reasons for contraindication to surgery (i.e., bleeding/clotting disorders, significant medical comorbidities)	Exclusion ‐ Anomaly unrelated to the MMC ‐ Severe kyphosis ‐ History of prior spontaneous preterm birth^*^ ‐ Short cervix on mid‐trimester transvaginal US screening ‐ Placental abruption ‐ Prior hysterotomy in active segment ‐ Current HIV ‐ BMI > 40 prior to pregnancy ‐ Other reasons for contraindication to surgery (i.e., bleeding/clotting disorders, significant medical comorbidities)

^*^Both history of prior preterm birth and gestational age of < 26 weeks at time of repair can be evaluated on an individual basis and appealed to the Fetal Oversight Committee for exception.

Since the MOMS trial, maternal laparotomy‐assisted fetoscopic MMC repair has gained significant popularity due to its non‐inferior outcomes for neonates compared to open uterus MMC repair and the significant maternal benefits for the index and subsequent pregnancies [9, 10]. More hesitancy exists for maternal laparotomy to fetoscopic MMC repair in the setting of higher BMI due to the longer operative times and the potential challenges of the fetoscopic approach versus open fetal surgery. Additionally, fetoscopic surgery for MMC repair takes significantly longer than open fetal surgery for MMC repair, and we sought to understand if BMI would further affect operative times. Given this, we sought to report and evaluate our pregnancy outcomes for maternal laparotomy to fetoscopic MMC repair in the setting of maternal pre‐pregnancy 35–40 compared to BMI < 35.

## 2 METHODS

This is a cohort study of patients that underwent laparotomy to fetoscopic MMC repair with birth occurring prior to December 2024. The study start date was January 2022, the first month in which our center moved the BMI criteria for inclusion from a pre‐pregnancy BMI ≤ 35 to a pre‐pregnancy BMI ≤ 40. This was determined by chart review of the patient's outside prenatal care records. All cases that went to the operating room for a planned fetoscopic MMC repair during the study window were included. The patients were determined to be either in the BMI 35–40 category or not based on the *pre‐pregnancy* BMI, not the BMI at the time of surgery, though the BMI at the time of surgery was reported. This study received Institutional Review Board (IRB) approval as part of an ongoing IRB‐approved trial evaluating clinical outcomes at our maternal fetal care center, Cincinnati Children's Hospital IRB #2021‐0075. This study was deemed to be exempt from patient consent.

The 60 cases performed from 2016 to 2022 were not included in this study as we were not routinely offering fetoscopic MMC repair for mothers with BMI ≥ 35 in this time frame. We elected to study only contemporary cases in this study to minimize effects from other surgical variables, including changes in the surgical technique (which did happen over the first 40–50 cases) as well as the learning curve and expertise developed over time. Particularly, we were concerned that if we included our early cases, we might falsely overestimate a protective effect of a higher BMI at the time of fetoscopic surgery, when in fact it was just related to the years of performance.

The surgical technique of maternal laparotomy to 3‐port MMC repair has previously been published by our group [11]. Briefly, a limited midline laparotomy is performed to expose or exteriorize the uterus. Using ultrasound, the placental edges are marked, and the fetus is positioned into an optimal operative area in a placenta‐free window. Three 10‐french fetoscopic Check‐Flo cannulas are inserted into the uterus sharply, with the first placed under US guidance and the subsequent two placed under direct fetoscopic visualization. Prior to placement of each trocar, a trans‐uterine, trans‐amniotic U‐shaped stitch is placed using 2‐O PDS suture around the area of planned trocar insertion to attach the membranes and to prevent tenting. The three trocars are used to triangulate the lesion. A portion of amniotic fluid is removed and replaced with warmed, humidified CO_2_. The fetus is given an intramuscular cocktail of weight‐based fentanyl, vecuronium, and atropine, and the placode is then dissected until completely de‐tethered. Next, two overlapping umbilical cord matrix patches are sutured over the neural tube defect for duraplasty. The skin is then closed primarily with a running suture if possible, and, if not, a thin Alloderm patch is used as a third patch. Fetal heart rate and serial Doppler assessment of the umbilical artery and ductus venosus are monitored throughout the case. Intra‐amniotic antibiotics are given at the beginning and end of the case and, at the conclusion of the repair, the gas is removed and replaced with warmed lactated Ringer's. Fibrin glue (Tisseel) is injected at the uterine port sites and the trans‐uterine trans‐amniotic sutures are tied to close the uterine defects from the cannulas. The uterus is returned to the abdomen, and the laparotomy incision is closed in layers. The surgery is performed under general endotracheal anesthesia and epidural anesthesia. In addition to inhaled anesthetic, a continuous magnesium sulfate infusion is used during surgery for tocolysis, with a 6‐g bolus given at uterine exposure and an initial infusion rate of 3 g/h that is then titrated as needed for the duration of surgery. Magnesium is continued upon conclusion of the case at a rate chosen by the on‐call Maternal‐Fetal Medicine physician.

The primary outcome was the gestational age at delivery between the two groups. Given that we were assessing non‐inferiority between surgery in BMI 35–40 and BMI < 35, a pre‐specified difference of no less than 1 week shorter gestational age at delivery in the BMI ≥ 35 was pre‐determined for non‐inferiority analysis, representing the minimum clinically acceptable difference in gestational age. Non‐inferiority would be concluded using bootstrapping to calculate 95% CI of the median for the BMI ≥ 35 group. If the lower bound of 95% CI of the median gestational age at delivery with BMI ≥ 35 was above the pre‐determined clinically acceptable difference in medians (median gestational age at delivery with BMI < 35 minus 1 week), non‐inferiority would be claimed. To further analyze the association of BMI and gestational age at delivery, linear regression was performed between BMI at the time of surgery and gestational age at delivery. Secondary outcomes planned were the operative time between the BMI ≥ 35 and BMI < 35 group, including the fetoscopic surgical time and the non‐fetoscopic surgical time to determine if there was a particular aspect of the surgery which was prolonged secondary to the higher BMI. Evaluation of non‐inferiority was planned for total operative time with a pre‐defined time of no greater than 30 min longer for total OR time with the higher BMI group and no greater than 15 min longer for fetoscopic time and non‐fetoscopic time each for the higher BMI group.

Additional secondary analyses included the risks of maternal and pregnancy complications between groups. Specifically, known complications of in utero MMC repair were evaluated, including preterm prelabor rupture of membranes (PPROMs), need for cesarean delivery, chorion‐amnion separation, maternal pulmonary edema, and any wound‐related complications, as defined by Clavien‐Dindo Classification [12]. Additionally, given that pulmonary edema and other maternal complications are rare outcomes, the rate of supplemental O_2_ usage at 24 h postoperatively and the length of hospitalization were compared between groups as a surrogate marker of maternal status postoperatively. Regarding neonatal findings, birthweight and cerebrospinal fluid (CSF) leakage at MMC repair site were compared.

Given the expected relatively low N with fetoscopic surgery for MMC, continuous variables were reported as median (IQR) and compared via Wilcox RankSum. Categorical variables were compared via chi‐squared or Fisher's exact test as appropriate. Non‐inferiority was compared between continuous variables as defined above. Data plots were created via MatLab.

## 3 RESULTS

From January 2022 to December 2024, a total of 38 patients underwent a maternal laparotomy to fetoscopic MMC repair. A total of 12 patients had a pre‐pregnancy BMI of 35–40 and 26 had a pre‐pregnancy BMI < 35. One patient in the BMI < 35 group had a fetoscopic port placed; however, prior to initiation of fetal surgical intervention, the fetus developed significant Doppler and FHR abnormalities and the surgery was aborted. The fetal status recovered, and the pregnancy continued with close observation and subsequent postnatal MMC repair. This patient is included in the demographical data but not in the operative and postoperative data due to a much shorter operation and the fact that fetal surgery was not performed. One additional patient in the BMI < 35 group had completion of fetoscopic MMC repair but subsequently required emergent cesarean delivery for fetal distress prior to leaving the operating room. This patient was included in all phases of the analysis as the surgery had been completed prior to delivery. There were no conversions to an open uterine approach, and no fetal or perinatal deaths occurred during the study timeline.

**FIGURE 1:**
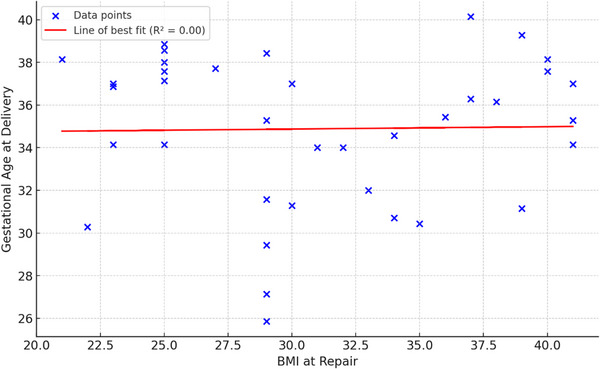
BMI is not associated with gestational age at delivery. Linear regression between patient BMI and gestational age at delivery revealed no correlation, with *R* ^2^ < 0.01.

Demographical factors between pre‐pregnancy BMI ≥ 35 and BMI < 35 groups were compared (Table 1). The only difference between groups was the BMI at the time of surgery (expectedly higher in the BMI ≥ 35 group). Regarding the primary outcome of gestational age at delivery, there was no significant difference between groups, though there was a non‐significant trend towards a later delivery gestational age in the higher BMI group with a median gestational age of delivery 36 2/7 weeks (IQR 34 3/7, 37 5/7) versus 35 2/7 weeks (31 4/7, 37 4/7), *p* = 0.256. Regarding the pre‐specified margin for non‐inferiority, the median minus 1 week value was 34 2/7 weeks. The 95% CI of the median for BMI ≥ 35 was 34 3/7 to 37 6/7 weeks, consistent with maternal laparotomy to fetoscopic surgery for MMC being non‐inferior in the setting of BMI ≥ 35. To further analyze BMI and gestational age at delivery, linear regression was performed and identified no relationship between BMI and gestational age at delivery (*R* ^2^ < 0.01, Figure 1).

**TABLE 1:** Pregnancy and demographic factors in the pre‐pregnancy BMI < 35 and pre‐pregnancy BMI 35–40 groups. Data are presented as median (IQR) or *N* (%) unless indicated otherwise.

	BMI 35–40 pre‐pregnancy *N* = 12	BMI < 35 pre‐pregnancy *N* = 26	*p*
Age	29 (26.5, 30)	28 (25, 32)	0.926
BMI at repair	39 (37, 40.5)	29 (25, 30)	<0.001
Gravida	2 (1, 3)	2 (1, 3)	0.946
Para	1 (0, 1)	1 (0, 1)	0.380
RV at evaluation, US (mm)	10.5 (8.5, 12.5)	11 (9.1, 14.0)	0.502
LV at evaluation, US (mm)	11.8 (9.0, 14.2)	11.0 (9.0, 14.0)	0.940
RV at evaluation, MRI (mm)	11.3 (8.0, 14.3)	11.5 (10.2, 14.0)	0.551
LV at evaluation, MRI (mm)	12.3 (9.4, 14.2)	11.8 (10.1, 14.0)	0.800
Myelocele/myeloschisis	2 (17%)	6 (23%)	0.349
Lesional level			0.714
Thoracic	0 (0%)	1 (4%)	
L1	0 (0%)	0 (0%)	
L2	3 (25%)	1 (4%)	
L3	2 (17%)	6 (23%)	
L4	1 (8%)	4 (15%)	
L5	3 (25%)	13 (50%)	
Sacral	3 (25%)	1 (4%)	
Gestational age at repair, weeks	25 3/7 (25 1/7, 25 5/7)	25 2/7 (25 0/7, 25 5/7)	0.931
Maternal Comorbidities			
CHTN	0 (0%)	2 (8%)	0.925
PreGDM	0 (0%)	0 (0%)	1
Maternal Race			1
Non‐Hispanic White	11 (92%)	24 (92%)	
Non‐Hispanic Black	1 (8%)	0 (0%)	
Hispanic	0 (0%)	2 (8%)	
Asian	0 (0%)	0 (0%)	
Other	0 (0%)	0 (0%)	
Smoking status			0.830
Never	10 (83%)	22 (85%)	
Current	1 (8%)	1 (4%)	
Quit before/during pregnancy	1 (8%)	3 (12%)	

Abbreviation: CHTN, chronic hypertension; GDM, gestational diabetes mellitus; GHTN, gestational hypertension; LV, left ventricle; MRI, magnetic resonance imaging; PreGDM, pre‐gestational diabetes; RV, right ventricle; US, ultrasound.

Regarding the pre‐specified margins for non‐inferiority surgical times, the median operative time plus 30 min was 277 min. This was not within the 95% CI of the median for BMI ≥ 35 (median 252 min, 95% CI 233–271 min), indicating non‐inferiority of operative time in the setting of BMI ≥ 35. Additionally, the overall operative time was not significantly different between groups (Table 2). The fetoscopic surgery time was also not inferior in the higher BMI group, as the median fetoscopy time plus 15 min was 193 min in the lower BMI group. This was not within the 95% CI of the median for BMI ≥ 35 (median 163 min, 95% CI 145–181 min), indicating non‐inferiority of operative time in the setting of BMI ≥ 35. However, there is a possible confounder that 0/12 patients in the higher BMI group and 5/26 in the lower BMI group required an Alloderm patch for skin closure. We did evaluate the “non‐Alloderm” and did not see a difference there either. There was a significantly higher non‐fetoscopic surgery time in the BMI 35–40 group (80 min, IQR 63, 91, vs. 59 min, IQR 55, 71, *p* = 0.014), indicating that the time under anesthesia not performing fetoscopic surgery is longer when the BMI is higher. This counterintuitive finding may be explained somewhat by the low N and the extremely variable fetoscopy time from case to case, which had a much wider spread than the non‐fetoscopic/non‐uterus time across groups, thus while the fetoscopic surgery time was actually *lower* in the higher BMI patients, due to the wide range, this was not a significant difference, whereas the non‐fetoscopic portion of the surgery was very consistent, and thus the difference seen *was* significant.

**TABLE 2:** Perioperative and Pregnancy outcomes in the pre‐pregnancy BMI < 35 and pre‐pregnancy BMI 35–40 groups. Data are presented as median (IQR) or N (%) unless indicated otherwise. CSF, cerebrospinal fluid; O2, oxygen; PPROM, pre‐term pre‐labor rupture of membranes.

	BMI ≥ 35 pre‐pregnancy *N* = 12	BMI < 35 pre‐pregnancy N = 26^a^	*p*
**Operative factors**			
Operative time	252 (232, 259)	247 (230, 264)	0.737
Excluding Alloderm patch	252 (232, 259)	240 (216, 270)	0.913
Fetoscopy time	163 (151, 177)	178 (158, 216)	0.265
Excluding Alloderm patch	163 (151, 177)	175 (152, 215)	0.426
Non‐fetal surgery time	80 (63, 91)	59 (55, 71)	0.047
Alloderm patch needed for skin	0 (0%)	5/25 (20%)	0.152
Length of stay	5 (5, 6)	6 (5, 6)	0.094
Conversion to hysterotomy	0 (0%)	0 (0%)	1
Completion of procedure	12 (100%)	25 (96%)	0.999
Pulmonary edema	1 (8%)	0 (0%)	0.648
Need for O_2_ support at ≥ 24 h postop	4/12 (33%)	5/25 (20%)	0.411
Readmission rate^b^	5/12 (42%)	9/26 (35%)	0.345
Venous thromboembolism	0 (0%)	0 (0%)	1

**Pregnancy outcomes**	*N* = 12	*N* = 25	
Gestational age at delivery	36 2/7 (34 3/7, 37 5/7)	35 2/7 (31 4/7, 37 4/7)	0.257
PPROM (< 37 weeks)	3 (25%)	6 (24%)	0.935
Chorion‐amnion separation	5 (42%)	13 (52%)	0.651
Any maternal wound complication^*^	3 (25%)	2 (8%)	0.320
Maternal complications			
GHTN	4 (33%)	2 (8%)	0.066
Preeclampsia	1 (8%)	0 (0%)	0.316
GDM	2 (17%)	8 (31%)	0.453
Route of delivery			1
Vaginal	5 (42%)	11 (42%)	
Cesarean	7 (58%)	14 (58%)	
Birthweight	2811 (2277, 3204)	2570 (1675, 3130)	0.262
Perinatal death	0 (0%)	0 (0%)	1
CSF leak at birth	0 (0%)	1/25 (4%)	0.942

^a^ All five maternal wound complications were categorized as small < 4 cm skin separations, which responded to conservative measures with the exception of one case of cellulitis responsive to antibiotics.

^b^ Readmission was defined as any overnight hospital stay within 30 days of surgery, for any indication, as per CMS guidelines.

Other outcomes between groups are presented in Table 2. There were no significant differences between groups, however the low number of patients prevented examination of non‐inferiority in categorical variables. Specifically, the postoperative length of stay, rates of pulmonary edema and need for supplemental oxygen at or beyond 24 h postoperatively were similar between groups. The rate of PPROM < 37 weeks (25% in the BMI ≥ 35 group vs. 24%, *p* = 0.935) or chorion‐amniotic separation (any separation noted, 42% vs. 52%, *p* = 0.651) were not different between groups, and the rate of vaginal birth was equivalent between groups (42% for both, *p* = 1). While there was not a significant difference in wound‐related complications between the groups (25% vs. 8%), there is a clear trend toward increased wound complications in the higher BMI group. All wound complications were either Clavien‐Dindo Classification Grade 1 or 2 (i.e., very small skin dehiscence managed conservatively ± short course of oral antibiotics without any additional imaging or interventions), and none required debridement, lancing or additional procedures. There was a high risk of postoperative readmission or observation in both groups (42% in the higher BMI group vs. 35% in lower BMI group, *p* = 0.346), and this was primarily driven by the aforementioned chorion‐amniotic separation rate, which is typically managed inpatiently in our practice. There was only one patient with findings concerning for CSF leak noted at delivery, and this was in the BMI < 35 group. Given that these evaluated outcomes from surgeries and deliveries occurred in 2022–2024, CSF diversion rates at 12 months are not compared due to low number of available cases, but thus far, 2/6 patients (33%) with pre‐pregnancy BMI ≥ 35 have required CSF diversion by 12 months of life. This is consistent with our previously published cohorts of fetoscopic MMC repair, but follow‐up studies are planned on differences, if any, in pediatric outcomes by maternal BMI.

## 4 DISCUSSION

The findings in this study suggest that maternal laparotomy to fetoscopic surgery for MMC repair is feasible in patients with pre‐pregnancy BMI of 35–40, with similar pregnancy outcomes as in those with BMI < 35. There was no increase in maternal or fetal complications in the BMI ≥ 35 group, and non‐inferiority was established regarding gestational age of delivery after surgery. These results indicate that BMI ≥ 35 alone should not preclude offering fetoscopic MMC repair if the patient is otherwise a good candidate. However, the longer non‐fetoscopic operative time observed in the BMI ≥ 35 suggests that habitus has some impact upon surgical time, and the non‐significant increase in wound complications postoperatively should not be trivialized.

There is little data available regarding fetal surgery outcomes in patients with Class 2 or even 3 obesity and even less data in the context of fetoscopic surgery in this population. A PUBMED literature review using the MESH terms “fetal surgery” AND “morbid obesity” yielded no relevant results and “fetal surgery” AND “obesity” yielded a total of only seven results. Of these, five were related to fetal surgery, one of which was related to fetoscopic surgery for TTTS, three were related to open fetal surgery for in utero repair of spina bifida, and one was a review of the 3 open fetal surgery cohort studies. This study is thus novel in that it is one of only two studies to evaluate the impact of maternal BMI upon fetoscopic surgery, one of only three studies to evaluate the impact of maternal BMI upon fetal surgery for MMC repair, and the only study to evaluate the impact of maternal BMI upon fetoscopic MMC repair. In the prior studies examining fetal surgery via hysterotomy for MMC repair, the impact of BMI upon outcomes was mixed. Moldenhauer et al [3] reported a longer operative time in the BMI ≥ 35 group without a difference in gestational age at delivery whereas Hilton et al [4] reported a 2 week earlier gestational age at delivery (32 weeks vs. 34 weeks) in patients with open MMC repair and BMI 35–40. This finding in the Hilton study was the impetus for our primary outcome to confirm non‐inferiority with gestational age at delivery. The Zurich group [13, 14] noted no differences in outcomes related to BMI. Similarly, Gebb et al [15] noted longer operative times with percutaneous surgery for fetoscopy for twins without an impact upon fetal outcomes. Our research aligns with these results, suggesting that, while the non‐fetoscopic portion of the case is longer related to maternal habitus, this does not confer an increased risk of complications. Our study goes further to be the first to establish non‐inferiority in regard to gestational age at delivery when comparing women with BMIs of 35–40 to those with BMIs < 35. While we only have 1 year of neonatal follow up on 6 of the patients with BMI ≥ 35, the 33% CSF diversion rate is consistent with our prior published series of maternal laparotomy to fetoscopic 3‐port MMC repair [16].

A particular concern of surgery in the setting of Class 2 or even 3 obese is related to the increased risks of longer operations and thus perioperative complications. This is a particular area of concern in the fetal surgery world, as fetoscopic surgery for MMC repair can take many hours to complete. These longer operative times may increase the risks of direct surgical complications such as infections, and complications such as pulmonary edema and atelectasis may also arise due to use of tocolytic medications both during and after these surgeries. In the MOMS trial [1], the rate of pulmonary edema was significantly increased with fetal surgery (6% vs. 0%, *p* = 0.03). In the report of the International fetoscopic neural tube defect repair consortium [17], surgery times were reported of 217.5 ± 84.17 min with fetoscopy compared to 78.5 min with MMC repair via hysterotomy, [1, 18] but the consortium identified equivalent rates of pulmonary edema (5% vs. 6%). In our higher BMI groups, our median operative time was similarly longer than what has been reported in MMC repair via hysterotomy (median of 252 min) and one patient experienced pulmonary edema postoperatively. Given that both the overall number of patients and the pulmonary edema rate are low, we also evaluated the use of any supplemental oxygen on postoperative day 1 or beyond, and these rates were similar between groups regardless of BMI.

One potential complication that should not be trivialized is the risk of a wound related complication. A unique aspect of a mid‐trimester fetal surgery with a large maternal laparotomy is related to the continued incisional tension after surgery. As the uterus grows, there is increased intra‐abdominal pressure [19, 20], and from 24 weeks to 40 weeks (virtually the entire postoperative time after an MMC repair) there is an almost exponential increase in intra‐abdominal volume, with a 61% percent increase by the end of pregnancy [21]. This increased incisional tension may theoretically increase the risk of wound dehiscence, particularly in the setting of obesity. Further complicating this is the significantly higher risk of infectious complications in obese patients after laparotomy [22]. When evaluating laparotomies in all patients stratified by BMI, the American College of Surgeries identified a specifically increased risk of wound infection and VTE (OR 1.24 and 1.39, respectively) in patients with BMI 35–40. While our patients did not experience VTE and there were no differences in wound related issues, it is fair to be concerned about the higher (albeit non‐significantly) rate of superficial wound/skin separations seen in the higher BMI group. Patients should be counseled preoperatively related to this potential risk, and wounds should be followed closely for signs of infection in the weeks after surgery. Furthermore, long term follow‐up is needed to assess the risk of hernia after maternal laparotomy to fetal surgery in this obese population.

As mentioned, the understanding of the impact of maternal obesity upon fetal surgery is poorly described, and longer term follow up is needed both on the pregnant patients that undergo these surgeries as well as the children that are experiencing longer anesthesia and operative times. However, by proving the non‐inferior nature of fetal MMC repair in patients with higher BMI's, this may open the door to other fetal surgeries being initially offered to not just patients with normal BMI, but also to patients with at least Class 2 obesity, which would be critical given obesity's association with congenital malformations [6, 7, 8]. If a broadening of patients receiving fetal surgery is considered, though, further research is warranted to develop postoperative care guidelines and to better describe the risks of adverse outcomes. For example, future research is needed regarding the risks and benefits of activity modification after fetal surgery. While ACOG recommends continuation of physical activity and encourages about 150 min of exercise weekly [23], it is not uncommon for patients to be instructed to drastically reduce their physical activities, potentially even to the point of bed rest, in an attempt to decrease the risks of preterm birth after a fetal intervention. This activity reduction is completely at the discretion of the physician, and there is no literature to support activity modification beyond that of fascial healing after surgery. When one considers that activity restriction is both associated with gestational diabetes mellitus [24] and increased risk of excessive weight gain during pregnancy [25], both of which increase adverse pregnancy outcomes, it is imperative to further study the impact of maternal activity after fetal surgery. Future studies should evaluate whether activity restriction after fetal surgery exacerbates GDM risk, particularly in obese patients who are already at higher risk of this complication. Concurrently, research is warranted to better establish postoperative care guidelines for obese and non‐obese patients.

The novel nature and rigorous evaluation of non‐inferiority speak to the strength of this study. Furthermore, this study evaluated numerous operative and postoperatively characteristics to attempt to evaluate potential subtle differences between laparotomy to fetoscopic MMC repair in BMI 35–40 versus BMI < 35. The use of a standardized surgical approach regardless of BMI ensures consistency and comparability of outcomes across all patients. Additionally, while 99 fetoscopic MMC repairs have been performed at our institution, only evaluating cases after adjustment in inclusion criteria to include patients with pre‐pregnancy BMI of 35–40 minimizes the potential for a confounding variable of a learning curve or changes in technique over time.

Though there are strengths, significant limitations must be acknowledged. First, the sample size was small, particularly in the BMI ≥ 35 group, limiting the power to detect differences in rare outcomes such as pulmonary edema or significant wound complications. Additionally, all fetal dysraphism repairs were done using a 3‐mini‐port approach and applying these results to non‐3‐port repairs may not directly translate. Finally, while this study focused on pregnancy specific outcomes to evaluate feasibility of the surgery, it did not evaluate longer‐term neonatal outcomes, such as rate of CSF diversion, and did not assess long‐term maternal outcomes such as rates of incisional hernia, both of which are critical for assessing the overall success of the procedure. Lastly, given that this was performed at a single high‐volume fetal center, while the results and repair are homogenous and consistent, these results may not directly translate to other centers.

## 5 CONCLUSION

Maternal laparotomy to fetoscopic surgery for in utero fetal MMC repair is feasible in patients with Class 2 obesity, with non‐inferior pregnancy length when surgery is performed in patients with a pre‐pregnancy BMI 35–40 compared to a BMI of < 35. Consideration should be given carefully to evaluate patients with BMI > 35 for possible in utero MMC repair.

## CONFLICT OF INTEREST STATEMENT

The authors declare no conflicts of interest.

## ETHICS STATEMENT

This study received IRB approval as part of our ongoing registry of clinical outcomes of patients treated at our maternal fetal care center, IRB# 2021‐0075.

## CONSENT

This study was exempt from patient consent per IRB review.

## Data Availability

The authors will provide de‐identified data upon reasonable request.
