# Correction to “Maternal laparotomy to fetoscopic myelomeningocele repair in patients with Class 2 obesity is safe and feasible”

**DOI:** 10.1002/pmf2.70180

**Published:** 2025-11-15

Forde, B., C.B. Stevenson, F.‐Y. Lim, M. Habli, D.N. McKinney, K.B. Markham, M. Hoffman, K.S. Nissen, E. Goetz, K. Bierbrauer, and J.L. Peiro, (2025). Maternal Laparotomy to Fetoscopic Myelomeningocele Repair in Patients With Class 2 Obesity is Safe and Feasible. *Pregnancy*, 1: e70078. https://doi.org/10.1002/pmf2.70078.

Charles B. Stevenson's name was listed incorrectly when this article was published online. His name has now been corrected.

We apologize for this error.

